# Encephalomyocarditis Virus in Non-Domesticated Species

**DOI:** 10.3390/pathogens14040397

**Published:** 2025-04-20

**Authors:** Remco A. Nederlof, Bon-sang Koo, Cecilia Sierra Arqueros, Leonor Natividad Camacho Sillero, Francis Vercammen, Jaco Bakker

**Affiliations:** 1 Independent Researcher, 2861 XZ Bergambacht, The Netherlands; 2 National Primate Research Center, Korea Research Institute of Bioscience and Biotechnology, Cheongju 28116, Republic of Korea; porco9@kribb.re.kr; 3 Department of Biomolecular Science, KRIBB School of Bioscience, University of Science & Technology, Daejeon 34141, Republic of Korea; 4 Veterinary Department, Selwo Aventura Zoo, Avenida Parque Selwo s/n, 29680 Estepona, Spain; csierra@selwo.es; 5 Selwo Marina Zoo, Parque de la Paloma s/n, 29630 Benalmádena, Spain; 6 Programa de Vigilancia Epidemiológica en Fauna Silvestre (PVE), Consejería de Sostenibilidad, Medio Ambiente y Economía Azul, Junta de Andalucía, 29002 Málaga, Spain; leonorn.camacho@juntadeandalucia.es; 7 Centre for Research and Conservation, Royal Zoological Society of Antwerp, K. Astridplein 20-26, B-2018 Antwerp, Belgium; francis.vercammen@kmda.org; 8 Animal Science Department, Biomedical Primate Research Centre, Lange Kleiweg 161, 2288 GJ Rijswijk, The Netherlands; bakker@bprc.nl

**Keywords:** EMCV, zoo, wildlife, myocarditis, virus, transmission, pathology, diagnosis, biosecurity, vaccination

## Abstract

Encephalomyocarditis virus (EMCV) causes sporadic and epizootic outbreaks among various domesticated and non-domesticated animal species worldwide. Although outbreaks are mostly reported in domestic pigs, mortality is reported in elephants, ungulates, nonhuman primates (NHPs), and rodents. Rats of the genus *Rattus* serve as primary reservoirs and vectors, but alternative infection routes have been proposed. Clinical disease is characterized by acute heart failure in most taxonomic groups, often culminating in rapid death. Due to the rapid progression of the disease, diagnostic confirmation is most commonly obtained postmortem. Pathological examination reveals interstitial lymphohistiocytic myocarditis and multiorgan congestion in most cases. EMCV is often demonstrated with RT-PCR or virus isolation techniques, but other methods, e.g., serology and immunohistochemistry, are available. The rapid progression of EMCV precludes effective therapeutic intervention, though agents such as interferon, verapamil, and curcumol have shown potential efficacy. Preventative strategies are crucial, emphasizing biosecurity measures to mitigate rodent contamination of feed and water. Inactivated vaccines have demonstrated protective efficacy in experimental models involving mice, pigs, and elephants, with analogous immunogenic responses observed in various zoological species. Live attenuated vaccines have conferred protection in pigs and NHPs, albeit with variable seroconversion rates in different species.

## 1. Introduction

*Encephalomyocarditis virus* (EMCV) is a non-enveloped, positive-sense, single-stranded RNA virus, belonging to the genus *Cardiovirus A* in the family *Picornaviridae* [1]. EMCV is primarily transmitted by rodent vectors, especially rats of the genus *Rattus* [2]. Infections are associated with sporadic and epizootic mortality events in domestic pigs (*Sus scrofa domesticus*) and zoological species around the world. It is considered to be an important pathogen of domestic pigs, and outbreaks are reported to have substantial health, welfare, and economic impacts [3,4,5,6,7,8,9,10]. Mortality rates approaching 100% are reported in suckling and pre-weaning piglets, whereas mortality is lower in post-weaning to adult animals. Subclinical infections are not often reported and may be underdiagnosed [3,7,11]. Infection results in rapid death due to myocarditis, particularly in piglets. Moreover, reproductive failure is reported in sows, which may manifest as abortion, stillbirth, and fetal mummification [12,13,14].

EMCV is considered to be zoonotic, although clinical disease is considered to be rare in humans [15,16]. The virus is reported worldwide, and the risk of exposure is considered to be high for people, particularly under unsanitary conditions. For example, a serological survey conducted in China reported a seroprevalence rate of 30.56% (1010/3305) among the general population [6].

Although pigs are the major species affected by EMCV, outbreaks have been reported in zoological institutions and wild animal populations since the discovery of the virus in the 1940s [17,18,19,20,21,22,23,24,25,26,27]. Infections have been reported in a wide range of mammalian taxa, but elephants and nonhuman primates (NHPs) are considered to be most susceptible to lethal infection [17,21,23,24,25,27,28,29,30,31]. EMCV cases in domestic and non-domestic animals may be solitary, but often occur in epizootic events characterized by rapid disease progression or sudden death in large numbers of animals [20,21,27].

Following infection, the virus enters through M cells of the intestinal Peyer’s patches [32]. It is likely that the virus replicates in macrophages at the entry site and subsequently disperses via the lymphatic system into the circulation. EMCV is primarily myocardiotropic, but may also be epitheliotropic to an extent [32]. Acute myocarditis with subsequent congestive heart failure and/or conductive abnormalities is thought to be the primary cause of death in most non-domesticated animals [18,23,33].

Historically, EMCV strains were subdivided into neurotropic and myocardiotropic variants, based on tissue tropism in mice [34]. These myocardiotropic variants are further subdivided into diabetogenic and nondiabetogenic strains, based on their ability to infect pancreatic b cells and induce type 1 diabetes in mice [35].

Three serotypes exist: EMCV-1, EMCV-2, and EMCV3 [1,31,36]. Eight EMCV-1 lineages, denoted A-H, and four evolutionary clades, designated I-IV, are recognized [37]. Lineages A, B, C, and G have been isolated from a range of domestic and zoological species other than rodents and pigs [23,30,37,38,39,40,41,42].

Diagnostic options for EMCV include virus isolation, reverse transcription polymerase chain reaction (RT-PCR), electron microscopy, genome sequencing, enzyme-linked immunosorbent assay (ELISA), serum neutralization (SN), hemagglutination inhibition (HI), and immunohistochemistry [17,26,27,28,31,43,44,45,46,47]. Blood and heart tissue are the samples of choice for the diagnosis of EMCV [44].

Several attempts have been made at developing a vaccine against EMCV, with variable success. To the authors’ knowledge, no commercially available vaccines are currently marketed. As a result, heavy emphasis should be placed on rodent control and appropriate disinfection practices.

This review aims to summarize the current literature on the occurrence, transmission routes, antemortem and postmortem disease characteristics, diagnostic procedures, treatment options, and prophylactic and biosecurity measures for EMCV in non-domesticated animals in captivity. It highlights knowledge gaps and research opportunities and aims to provide veterinary and animal management staff with relevant information for the management of EMCV in a zoological setting. To identify all the relevant literature, we conducted a search for books, book chapters, and peer-reviewed publications in academic literature databases, such as PubMed and Google Scholar.

## 2. Transmission

EMCV is primarily transmitted via the fecal–oral route [39]. As a small, non-enveloped virus, EMCV is stable in the environment and may remain infectious for days in the environment [11].

### 2.1. Rodents

In most outbreaks, the ingestion of food, water, or carcasses contaminated with rodent feces has been proposed as the most likely source of infection [11,14,17,24,44]. Seasonality has been reported in farmed pigs and zoo animals, with incidence peaks occurring during the colder months of the year. It has been proposed that this reflects the tendency of rodents to shelter indoors during the cold season [3,17,21,24,25,26,28,44].

EMCV has been successfully isolated from a range of wild rodent species, e.g., mice (*Mus* spp.), rats (*Rattus* spp.), *Mastomys* sp., dormice (*Myoxus glis*), cotton rats (*Sigmodon hispidus*), water rats (*Hydromys chrysogaster*), and spiny rats (*Proechimys guayennsis*) [19,25,37,38,48,49,50,51,52]. Rodents belonging to the family *Muridae* and genus *Rattus* are considered to be primary EMCV reservoirs [13,14,25,31,38].

All phylogenetic lineages demonstrate links between rodent-associated EMCV and EMCV isolated from non-rodent hosts in the geographic area [37]. These links include porcine viruses in Belgium, Greece, and Italy clustering with those from brown rats (*Rattus norvegicus*), and porcine viruses in Cyprus and Italy clustering with those isolated from *Apodemus* sp. [37]. A role for *Mastomys* sp. rodents was implied in an outbreak in wild African elephants (*Loxodonta africana*) [21]. At the time, live EMCV could not be isolated from rodents, but a seroprevalence of 25.2% across rodent species was reported, with the highest (95%) seroprevalence reported in *Mastomys* sp. Subsequent RT-PCR tests confirmed the presence of EMCV nucleic acid in 60% (6/10) of the evaluated *Mastomys* sp. individuals [21]. Recent phylogenetic analysis confirms that three isolated *Mastomys*-associated viruses are highly related to two strains isolated from an elephant outbreak [37]. Moreover, EMCV shed by rats, trapped near elephant enclosures in France, was demonstrated to be 98.1–99.9% identical to virus isolates obtained from an elephant who succumbed to EMCV infection [39]. In Belgium, a strain isolated from a deceased Malayan tapir (*Tapirus indicus*) had 100% sequence alignment with a simultaneously circulating porcine isolate, suggesting a common source [53]. Links between rodents and other species are observed within the same geographical area, but also in other parts of the world [37]. This may be the result of the wide geographic ranges of invasive rodent species that are facilitated by human-mediated movement [54].

Experimental infections of mice and rats suggest that mice are more susceptible to EMCV-associated encephalitis [55]. Experimentally infected mice developed rear and, sometimes, front limb paralysis. Over half of the animals died between 3 and 5 days following infection [24]. Fecal–oral EMCV transmission occurs from 2 to 29 days post-infection in mice. Moreover, EMCV is demonstrated to persist in thymic macrophages, which may allow for latent infection with subsequent shedding under stressful conditions [2,56]. Rats, however, were resistant to disease following experimental infection in one study [2]. Although neutralizing antibodies were produced, titers were low and delayed after infection. As a result, viral excretion may occur for several weeks. The virus has been isolated from rat feces 29 days post-challenge, and is demonstrated to persist in the rat population by rat-to-rat transmission [2]. EMCV was consistently isolated from the thymus and Peyer’s patches 62 days post-infection in rats, suggesting persistent infection [2].

### 2.2. Other Vectors

Other vectors and reservoir species have been proposed. EMCV has been reported to circulate in wild boar (*Sus scrofa*) populations in Europe and Asia [3,49,57,58]. It is uncertain whether boar may serve as a reservoir species, or whether the high reported prevalence of EMCV is a result of a constant infection pressure [3,49]. Nevertheless, wild boars are considered to be invasive species in the Americas, and their potential role in EMCV dissemination should be investigated further [59].

Similarly to rodents, non-rodent survivors of infection may become carriers. In experimentally infected piglets, dexamethasone treatment induced short-lived (1–2 days) virus excretion after initial virus excretion subsided [60]. Under stressful conditions, carrier animals may resume shedding of EMCV.

Eastern bent-wing bats (*Miniopterus fuliginosus*) are implied as natural EMCV reservoirs in Asia, as the virus has been demonstrated by RT-PCR from guano in caves inhabited by these animals [61]. Moreover, EMCV has been isolated from various arthropods, including mosquitoes, ticks (*Ixodes petauristae* and *Haemaphysalis spinigera*), parasitic crustaceans (*Porocephalus armillatus*), and rodent mites (*Laelaps muricola*) [37,50,62,63,64,65]. Although transmission studies using mosquitoes (*Ades aegypti*) were unsuccessful, the role of vector-borne EMCV transmission has been understudied [64,65]. The importance of other potential vector species in the epidemiology of EMCV requires further research.

Considering that EMCV may remain infectious for days in the environment, personnel and materials should be regarded as potential fomites [17].

### 2.3. Intraspecies Transmission

Although transmission via the fecal–oral route from rodents to non-rodent mammals is considered to be the most important route, direct transmission between pigs has been reported. Both vertical and horizontal transmission may occur, but likely play a small role in EMCV epidemiology [66,67,68]. More research is necessary to determine the role of intraspecies transmission during outbreaks in zoological institutions.

### 2.4. Predation

Transmission by feeding on infected carcasses has been proposed. Following the death of an African elephant in a zoo, the carcass was fed to a group of lions (*Panthera leo*). Subsequently, 20 lions died within a short timeframe and had postmortem lesions suggestive of EMCV infection. Virus isolation was, however, not performed in these animals [20]. In contrast, no transmission through predation was observed in three lions that fed on a confirmed elephant case during an outbreak in the Kruger National Park, as indicated by a lack of seroconversion three months after potential exposure. It may, however, be the case that infection did not occur because the lions did not consume heart tissue, which contains the highest viral loads [21].

## 3. Clinical Disease

Across EMCV variants and animal taxa, clinical signs are mostly nonspecific or are related to acute heart failure. Clinical signs are reported in 69.2% (72/104) of reported cases, and most frequently consist of lethargy (41.2%; 14/34), hyporexia or anorexia (20.6%; 27/34), recumbency (17.6%; 6/34), colic (17.6%; 6/34), dyspnea (17.6%; 6/34), and ataxia (17.6%; 6/34) (see Appendix A, Table A1).

### 3.1. Artiodactyls

A mortality rate of 100% is reported in non-domestic artiodactyls, with all artiodactyls showing clinical signs of disease before death (See Appendix A, Table A1). A pygmy hippopotamus (*Choeropsis liberiensis*) is reported to have died due to EMCV infection after demonstrating marked lethargy and inappetence for one day [27]. Lethargy and anorexia are also reported in semi-wild boar in China, which concurrently demonstrated tachypnea and ataxia [58]. An oryx–addax antelope hybrid (*Oryx* sp. × *Addax nasomaculatus*) reportedly died due to EMCV infection following a brief period of anorexia and fever [20]. Colic and recumbency were reported in all but one alpaca (*Lama pacos*) (5/6) during an outbreak in New South Wales. Two of these animals were also reported to be ataxic [44]. Seroconversion, but no signs of clinical disease, are reported in a tule elk (*Cervus canadensis nannodes*) and white-tailed deer (*Odocoileus virginianus*) [69].

### 3.2. Perissodactyls

EMCV infections have been reported in tapirs, with marked differences in clinical outcomes. On the one hand, seroconversion without concurrent clinical disease is reported in a South American tapir (*Tapirus terrestris*) [69]. On the other hand, EMCV infection resulted in the death of a five-month-old Malayan tapir. This animal was found dead in the enclosure without premonitory clinical signs [53]. Acute death due to infection is described in rhinoceroses (*Rhinocerotidae*), but no details were provided [70]. These cases highlight the lack of knowledge about the pathogenicity of the virus in closely related species. Further research should be conducted to elucidate the underlying factors predisposing to higher morbidity and mortality in some individuals and species.

### 3.3. Nonhuman Primates (NHPs)

NHPs are highly susceptible to EMCV infection. Mortality is reported in all NHP families, ranging from lemurs to great apes (see Appendix A, Table A1). Following experimental infection, the disease ranged from asymptomatic infection to lethal disease in one report [24]. Here, mortality was observed in rhesus macaques (4/13), grivets (*Chlorocebus aethiops*) (3/5), and hamadryas baboons (2/4) [24]. Naturally infected bonobos only displayed tachypnea and dyspnea before succumbing to the infection within 24 h after the onset of clinical signs [23]. Death with only a short history of hyporexia is described in a Sumatran orangutan (*Pongo abelii*). Interestingly, a female conspecific did not develop clinical signs, but exposure to the virus was confirmed by means of hemagglutination-inhibition testing [18]. In Barbary macaques (*Macaca sylvanus*), only lethargy and weakness have been reported antemortem [28].

Death without premonitory clinical signs has been reported in common marmosets (*Callithrix jacchus*), squirrel monkeys (*Saimiri sciureus*), de Brazza’s monkeys (*Cercopithecus neglectus*), Barbary macaques, hamadryas baboons, mandrills (*Mandrillus sphinx*), orangutans (*Pongo* sp.), and ring-tailed lemurs (*Lemur catta*) [17,20,24,27,30,31]. It has been proposed that the rapid death observed in apes, and likely other species, is the result of severe virus-induced and/or inflammatory damage to the cardiac conduction system, which has been experimentally confirmed in mice [23,33]. The resulting disruption in electrical transmission subsequently causes arrhythmias, asystole, and/or congestive heart failure [18]. Similarly, tachycardia and ECG changes associated with myocarditis or myocardial infarction have been observed in experimentally infected baboons [24]. It is highly likely that the same or similar mechanisms result in the rapid death observed in non-NHP species.

### 3.4. Elephants

Grobler et al. (1995) described an outbreak in Kruger National Park from 1993 to 1994, which resulted in the death of 64 free-ranging African elephants over the course of nine months. Interestingly, 83% (53/64) of cases occurred in adult bulls. Serological surveillance was performed, which indicates that EMCV in wild African elephants has an approximate fatality rate of 2.6–10.6% [21]. In another case report, one adult African elephant presented with apathy and tremors of the distal end of the trunk for one hour before collapsing and dying [71]. An experimental study reports malaise and ECG abnormalities in an experimentally infected African elephant. Here, two out of six animals died without premonitory clinical signs [29]. Death without antemortem clinical signs has also been reported following natural infection [25].

EMCV has been isolated from a single Asian elephant (*Elephas maximus*). This animal died following a 24 h period of severe colic, vomiting, diarrhea, and tenesmus. It is, however, likely that some of these clinical signs are related to the severe enteritis that was observed during postmortem examination, which is likely to be unrelated to the EMCV infection [20]. It can, however, not be excluded that an underlying process predisposed this animal to both severe enteritis and EMCV infection.

### 3.5. Felids

Confirmed cases of morbidity or mortality due to EMCV infection are rare in felids. However, deaths due to EMCV infection are reported in South China tigers (*Panthera tigris*). Clinical signs were nonspecific and consisted of reduced appetite and lethargy [72]. Seropositivity in the absence of clinical signs is reported in two Asiatic lions (*Panthera leo leo*), a black leopard (*Panthera pardus*), a clouded leopard (*Neofelis nebulosa*), and a cheetah (*Acinonyx jubatus*) following an outbreak in a zoo [69]. During an outbreak in a zoological institution, carcasses of African elephants that succumbed to EMCV were fed to lions (*Panthera leo* sp.). Several of these animals became anorectic and lethargic, and 20 animals died within a 26-day timeframe. EMCV infection was not confirmed in these lions, as no attempt at virus isolation was performed. Nevertheless, the history of feeding on infected carcasses and findings on postmortem histological examination are suggestive of EMCV infection [20].

### 3.6. Rodents

Clinical disease progression may vary in mice and rats from asymptomatic infection to encephalitis, limb paralysis, myocarditis, reproductive disorders, and/or type 1 diabetes [56,73,74,75]. Neurological clinical signs are more frequently reported in rodent species, possibly as a result of slower disease progression. In a capybara (*Hydrochoerus hydrochaeris*), a course of disease lasting five days has been described, characterized by intermittent hindlimb ataxia and lethargy. Clinical signs progressed in 24 h to hindlimb ataxia and paresis, nasal discharge, and bradycardia [43]. In this animal, radiographs were performed, revealing a generalized opacity of the right lung, likely indicative of pulmonary effusion [43]. Similar clinical signs are reported in another capybara. This animal demonstrated weakness, weight loss, and hemiparesis. An ECG was performed, which revealed the absence of P-waves and widened QRS complexes. Blood biochemistry demonstrated an elevated creatinine phosphokinase of 28,080 U/L [69].

### 3.7. Other Species

The virus was isolated from the heart of a wild cottontail rabbit (*Sylvilagus* sp.) following an outbreak in a zoo. Unfortunately, it is unknown whether this animal had any clinical signs or whether it was found dead or caught alive [69].

Raccoons (*Procyon lotor*) experimentally challenged with 0.5 mL of 1 × 10^6.5^ TCID_50_ EMCV intramuscularly (IM) (*n* = 1) or orally (*n* = 6) demonstrated low susceptibility to infection. No clinical disease was reported in either group [76].

Two Goodfellow’s tree-kangaroos (*Dendrolagus goodfellowi*) infected with EMCV did not show clinical signs prior to death. EMCV was isolated from the liver of both animals and the heart of one [27].

## 4. Postmortem Observations

An overview of reported gross pathological lesions is provided in Appendix A, Table A2. Reported lesions are interpreted in varying degrees in the literature used. Consequently, the columns of Table A2 may contain terminology with partially overlapping definitions. A lack of more comprehensive descriptions in the source literature precludes further interpretation of lesions and subsequent merging of the table columns.

Postmortem macroscopic lesions are present in most cases. However, a De Brazza’s monkey, a South China tiger, and an elephant who died peracutely were described to be free of gross pathological lesions upon postmortem examination in spite of successful virus isolation [20,21,72]. Lesions most frequently occur in the cardiovascular and pulmonary organ systems, and only 8.2% (12/147) of animals with reported lesions did not have any in the cardiovascular or pulmonary system (see Appendix A, Table A2) [17,21,28,29,72]. The most commonly reported lesions across animal taxa include free fluid in the pericardial sac (58.2%, 78/134) and thorax (40.2%, 80/209), pulmonary edema (43.8%, 84/192), pulmonary congestion (39.6%, 76/192), and the presence of pale foci on the myocardium (28.6%, 44/154). Histopathological lesions are reported in separate paragraphs following the macroscopic lesions.

### 4.1. Cardiovascular System

Systemic manifestations of congestive heart failure, including abdominal and pericardial effusion, are frequently reported across taxonomic groups. This fluid is reported to be clear or hemorrhagic and may contain fibrin clots [27,53]. Pericardial effusion is even more commonly reported (58.2%, 78/134), particularly in alpacas, NHPs, and rodents [17,24,26,28,43,44,69]. Other cardiac lesions include the presence of pale foci in the myocardium, which is reported in 28.6% (44/154) of cases (see Appendix A, Table A2). These foci may range in morphology from circular areas to large streaks [28,44,53]. In some cases, white foci may also be present on the endocardium and epicardium [21]. Petechial hemorrhages and/or ecchymoses are reported in the hearts of elephants, NHPs, and a South China tiger [17,20,27,72]. Cardiomegaly is reported in 8.5% (15/176) of cases and mostly occurs in crested porcupines (*Hystrix cristata*), capybaras, African elephants, NHPs, and a South China tiger (see Appendix A, Table A2). Infrequently, the consistency of the heart, in particular the left atrium, is described to be decreased in African elephants (20%, 4/20) [20,71]. In a large outbreak in hamadryas baboons, suggestive cardiac lesions were observed during the necropsy of 27 deceased animals. Lesions suggestive of myocarditis, as well as hydropericardium and mild cardiomegaly, were reported in this group. Virus isolation and viral genome sequencing were conducted in only one animal, which confirmed the infection [30].

The presence of focal, multifocal, or diffuse interstitial myocarditis is commonly reported in a wide range of species. White macroscopic foci may contain myocardial degradation and necrosis upon histological examination [18,21,27,28,32,43,44,71]. Inflammatory infiltrations are frequently reported, are often primarily lymphocytic and/or histiocytic, and may contain limited numbers of neutrophils and eosinophils [17,23,24,25,26,28,30,43,44,53,72]. In a Sumatran orangutan, inflammatory infiltrates are described to occur mostly in the cardiac conduction system [18]. Inflammatory infiltrates are also reported to be present in the epicardium of rhesus macaques, grivets, and hamadryas baboons [24]. Focal mineralization is reported in a deceased capybara, pygmy hippopotamus, and a Malayan tapir [27,43,53]. Calcifications are likely to be of the dystrophic type, which occurs secondary to myocardial necrosis [77]. Diffuse lymphangiectasia and perivascular hemorrhages with necrotizing vasculitis are described in deceased NHPs [17]. Hemorrhages are also reported in the endocardium, myocardium, epicardium, and epicardial adipose tissue of African elephants, as well as thrombosis and hemorrhages in other organs [21,25].

### 4.2. Pulmonary System

Pulmonary lesions are frequently described in most taxonomic groups, and mostly consist of pulmonary edema (43.75%, 84/192) and congestion (39.6%, 76/192) (see Appendix A, Table A2). The presence of, sometimes bloody, foam in the trachea is reported in NHPs, alpacas, an oryx-addax antelope hybrid, and a Malayan tapir, and is indicative of pulmonary edema [18,20,26,44,53]. Pulmonary emphysema is reported in only 2.5% (5/203) of cases and is only reported in a South China tiger and in ring-tailed and white-fronted (*Eulemur albifrons*) lemurs [17,72].

Interstitial pulmonary edema is frequently reported, mostly in NHPs and elephants [21,26,29]. Pulmonary congestion is described to be present histologically in a Malayan tapir, African elephants, alpacas, and crested porcupines [21,28,44,53]. A single case report also describes the presence of diffuse pulmonary atelectasis in a capybara [43].

### 4.3. Hepatobiliary System

Hepatic changes are observed occasionally, primarily consisting of hepatic congestion (8.7%, 17/196). Less frequently, hepatomegaly is reported (5.6%, 11/196) in African elephants, chimpanzees, rhesus macaques, a common marmoset, and an orangutan [17,18,20,21,26,71]. A zonal liver pattern is reported in two alpacas, and the presence of multifocal areas of pallor is reported in a Malayan tapir and an oryx-addax antelope hybrid [20,44,53]. Jaundice is uniquely reported in a single South China tiger, and, according to Liu et al. (2013), is likely incidental [72].

Centrilobular degeneration or necrosis is described sporadically in rhesus macaques, an African elephant, and a Malayan tapir [21,26,53]. Moreover, sinusoidal congestion is reported in alpacas, an African elephant, and rhesus macaques [21,26,44]. Uniquely, in rhesus macaques, hepatic lipidosis and lymphocytic portal hepatitis are reported in conjunction with congestion and hepatocellular necrosis [26].

### 4.4. Nervous System

Alterations in the nervous system are rarely reported, but macroscopically visible congestion of the meninges is reported in 5.6% (11/196) of cases and is likely secondary to congestive heart failure. One must keep in mind, however, that examination of the nervous system is not always performed during postmortem examination. Hence, these changes likely occur more frequently than reported.

Histologically, cerebral edema and hyperemia are reported in deceased rhesus macaques, grivets, and hamadryas baboons [24]. Central nervous system lesions reported in other NHPs include cerebral congestion, perivascular meningeal hemorrhage, satellitosis, and neuronal necrosis. A lack of positive signal on immunohistochemistry suggests that these lesions were secondary to anoxia, likely as a result of congestive heart failure [17]. Similarly, cerebral oedema is reported in African elephants [21]. Inflammatory infiltrates may be present concurrently. Perivascular lymphohistiocytic infiltrates are described in rhesus macaques, grivets, and hamadryas baboons, and multifocal lymphocytic meningoencephalitis is reported in six crested porcupines [24,28]. In alpacas, perivascular infiltrates are described to be neutrophilic and may occur in the cerebrum and adjacent leptomeninges [44].

### 4.5. Gastrointestinal System

Pathological changes of the gastrointestinal tract are rarely reported. Enteritis is reported in 2.0% (4/203) cases, specifically, in an Asian elephant, a Barbary macaque, and a ring-tailed and white-fronted lemur [17,20]. However, it is quite likely that enteritis is an incidental finding in these cases. The catarrhal enteritis reported in these NHPs was attributed to the presence of gastrointestinal nematodes that were reportedly present in this animal collection [17]. Therefore, it is quite likely that all enteritis cases are incidental findings. Congestion of the gastrointestinal tract, most likely secondary to congestive heart failure, is reported in two out of six alpacas and a Malayan tapir [44,53]. In these two alpacas, the gastrointestinal contents were also reported to be hemorrhagic [44].

Histologically, monocellular infiltrates are reported in the small intestinal epithelium of a bonobo [23]. Enteritis reported in a Malayan tapir was characterized by lymphocytic and plasmacellular infiltrates in the jejunal lamina propria [53]. Although these animals died due to EMCV infection, it is likely that the reported enteritis is incidental.

### 4.6. Lymphatic System

Lymphadenomegaly (2.0%, 3/154) and congestion of the mesenterial lymph nodes (1.5%, 3/196) are the only reported changes to the lymphatic system (see Appendix A, Table A2). Lymphadenomegaly is reported in two African elephants and a common marmoset [17,20,71]. These findings may be incidental, but could reflect the route that the virus takes after replicating in intestinal Peyer’s patches. Congestion of the mesenteric lymph nodes is reported in a single African elephant, an Asian elephant, and a Malayan tapir [20,53,71]. It is likely that this congestion is secondary to congestive heart failure.

Gross pathological changes of the spleen are rarely reported. Splenomegaly (2.0%, 4/196) and splenic congestion (1.5%, 3/203) are reported in elephants and various NHP species [18,20,27]. Splenic hemorrhage is reported in a single African elephant [20].

### 4.7. Renal System

Renal lesions are primarily confined to congestion (2.0%, 4/203), pallor of the renal cortex (1.5%, 3/196), and hemorrhages (1.0%, 2/196) (see Appendix A, Table A2). In alpacas, diffuse congestion is reported in all animals (*n* = 6) and is likely a consequence of congestive heart failure [42].

Histologically, diffuse congestion is reported in all six alpacas and is likely a consequence of congestive heart failure [44]. Mononuclear infiltrates in the kidneys are reported in a bonobo, and mild chronic tubulointerstitial nephritis is described in a capybara [15,41]. It is unclear whether these findings are incidental, but they appear to be quite rare regardless.

## 5. Diagnostic Methods

The virus can be demonstrated with virus isolation followed by (immune) electron microscopy, ELISA, genome sequencing, or RT-PCR. Moreover, RT-PCR may be used as a stand-alone technique, and immunohistochemistry, serum neutralization (SN), and hemagglutination inhibition (HI) assays may be used to demonstrate the presence of virus components in a sample [17,26,27,28,31,43,44,45,46,47].

### 5.1. Virus Isolation

EMCV is traditionally demonstrated by virus isolation from heart, brain, or splenic tissue [17,27]. Following virus isolation, the virus may be determined through (immune) electron microscopy, ELISA, genome sequencing, or RT-PCR [17,28,31].

### 5.2. RT-PCR

Nowadays, RT-PCR is considered to be the method of choice to demonstrate the presence of EMCV in a sample [43]. Reliable (q)RT-PCR assays yield results within a few hours, only require small samples, and can be used with suboptimal, e.g., autolyzed, samples [44].

The tissue sample used for RT-PCR is of great importance for the diagnostic accuracy of the test. Overall, heart tissue is considered to be the best sample for the detection of EMCV RNA, as EMCV demonstrates clear cardiac tissue tropism, resulting in high cardiac virus loads. The diagnostic sensitivity of brain tissue likely depends on whether there are lesions in the brain [45]. In alpacas, qRT-PCR was used to demonstrate the presence of EMCV in various samples. All examined samples were positive, and include aqueous humor, serum, EDTA- or heparin-treated blood, nasal swabs, oral swabs, rectal swabs, vaginal swabs, heart, brain, liver, lung, kidney, or mesenteric lymph node tissues. The lowest cycle threshold (Ct) values, corresponding to the highest viral loads, were reported for all types of blood samples and heart tissue samples (average Ct value: 19.28 (*n* = 2)). In the blood samples, the reported average Ct values are, respectively, serum 17.28 (*n* = 4), heparin-treated blood 19.17 (*n* = 2), and EDTA-treated blood 19.86 (*n* = 2). The highest average Ct values, corresponding to the lowest tissue levels of viral RNA, are reported for nasal (33.14 (*n* = 2)), oral (35.86 (*n* = 2)), and rectal (30.63 (*n* = 2)) swabs [44]. Blood samples, as well as pericardial fluid, were also determined to be suitable for RT-PCR in bonobos [23]. In an African elephant, EMCV was demonstrated by RT-PCR in heart, kidney, liver, and blood samples [25].

In an EMCV outbreak in 27 hamadryas baboons, RT-PCR using EMCV-1 specific primers was negative for myocardia of all (*n* = 18) tested animals. However, infection was confirmed by means of virus isolation and genome sequencing in one animal (1/1). It is likely that genetic differences between the strains used for the development of the primers and the novel lineage G strain resulted in false negative results [30].

Novel PCR techniques have recently been developed to improve diagnostic sensitivity and specificity. A RT-PCR with SYBR Green has been developed to detect and quantify EMCV in porcine tissues. This technique uses two primers specific for the highly conserved 3D gene. This PCR assay was demonstrated to detect EMCV titers at least 10^4^ times smaller than the routine assay [47].

A fluorogenic probe-based duplex real-time RT-PCR has been developed to detect strains previously undetected by older probes. This technique was 100-fold more sensitive than virus isolation in cell culture, and successfully detected Australian strains that could not be detected with the use of previously published reagents [45].

Even more recently, a reverse transcription-polymerase amplification assay (RT-RPA) combined with a lateral flow biosensor (LFB RT-RPA) or combined with the fluorescence detection platform (qRT-RPA) was developed. The RPA is designed to amplify the 3D gene, which is also amplified with the traditional qRT-PCR technique. A 100% diagnostic agreement was reported between qRT-RPA and qRT-PCR, and a 97.83% agreement between LFB RT-RPA and qRT-PCR. Although the novel qRT-RPA performed similarly to the established qRT-PCR, this technique is more rapid and may prove to be more suitable for field application in the future. A benefit of the LFB RT-PRA is the increased sensitivity over the qRT-PCR. None of these novel assays exhibited cross-reactions with other tested swine pathogens. Although these techniques were highly sensitive for EMCV lineage 1 strains, few nucleotide mismatches were reported for strains belonging to lineages 2, 3, 4, and 5. This may result in a reduced diagnostic accuracy [46].

It is important to note that multiple genetically distinct lineages may circulate in an area at any given time [40]. RNA replicase has a high mutation rate of approximately one mutation per replication cycle, with multiple replication cycles occurring in a single infectious cycle [78]. As a result, new EMCV strains may emerge rapidly and exist concurrently in endemic areas. Novel strains may differ in pathogenicity and transmission characteristics but may also escape detection by routine PCR techniques. Consequently, a negative PCR should be interpreted with caution, and concurrent use of other diagnostic techniques is advised.

### 5.3. Other Methods

In tissue samples, immunohistochemistry using the monoclonal 3E5 antibody may be used to demonstrate the presence of EMCV [17,23,26].

Serology is often of little diagnostic value, as animals frequently succumb before antibodies against the virus can be demonstrated in the blood. However, in some cases, death occurs only after a longer course of disease, in which case antibodies may be of diagnostic value [18,79]. Both SN and HI assays may be appropriate to demonstrate the presence of EMCV antibodies in serum [18,80].

## 6. Intervention Strategies

### 6.1. Therapeutics

Treatment of EMCV is often impossible due to the rapid progression of the disease. Moreover, the disease is relatively unknown, and appropriate diagnostic techniques are often not utilized until the late stages, if at all, in live animals, which may result in delayed treatment. In spite of the absence of a specific antiviral treatment, symptomatic treatment may be implemented when clinical signs suggestive of EMCV occur. Treatment may be attempted in animals with moderate clinical signs. In most cases, however, euthanasia should be strongly considered, as the prognosis of animals in congestive heart failure is poor.

Treatments have been attempted previously but have rarely been effective. For example, during a five-day course of disease in a captive capybara, treatment was attempted with unreported dosages of amoxicillin–clavulanic acid and meloxicam. Although mobility and appetite improved initially, the animal was found deceased on day five [43]. Moreover, multiple elephants have been treated with fluid therapy and unspecified antibiotic, analgesic, and antispasmodic therapy. Nevertheless, these animals still succumbed to the infection [20].

Only three EMCV-specific experimental treatments are reported.

Interferon was assessed in vivo in Yellow baboons (*Papio cynocephalus*) (*n* = 16). The animals were challenged with 10^4^–10^8^ PFU of virus subcutaneously. For five days, interferon was injected IM twice daily at doses of 3 × 10^6^ units. All treatment schedules prevented death, even in cases where treatment was started 24 h post-infection. The single administration of interferon at the same time as 10^5^ PFU of EMCV fully prevented viremia and disease in one animal (*n* = 1/1) [81]. In contrast with these findings, it is described that the structural EMCV protein V2 plays an important role in the evasion of the type I interferon signaling pathway, and, thus, constitutes a mechanism for EMCV to evade the host antiviral response [82]. Future research should be conducted to shed more light on the interplay between EMCV and interferon, and to evaluate interferon as a potential therapeutic option.

Verapamil, a calcium channel blocker, has been suggested to ameliorate cardiovascular clinical signs and reduce virus-associated inflammation and necrosis in experimentally infected mice [83]. However, no reports exist on its usage outside a laboratory setting for the treatment of EMCV.

Curcumol is a compound that is extracted from the roots of curcuma. It has been demonstrated that curcumol has anti-EMCV activity in vitro, and that it effectively reduces the viral load and maintains normal cell physiology, possibly due to an upregulation of IFN-b [84]. No in vivo studies have been performed. Therefore, more research should be conducted before this treatment can be implemented in practice.

Although these last three treatments are suggested to be effective, more research is required to better understand their efficacy and safety profiles in a wider range of species before these treatments can be implemented in veterinary practice.

### 6.2. Biosecurity

Measures aimed at reducing the incidence of EMCV often include rodent control programs. Rodent control programs have been successfully implemented during outbreaks and may include the use of rodent bait and trapping of wild animals [17]. Enclosures may be modified to deter rodent vectors from gaining entry. Moreover, food should be carefully stored and protected from contamination with rodent excreta. Uneaten food should be removed promptly from enclosures, and it is recommended to restrict feeding of animals to daylight hours in order to reduce the potential contact time of rodents with the food.

Although EMCV infection was not confirmed in lions that were fed on an EMCV-positive elephant, it is advisable not to use meat derived from suddenly deceased animals until EMCV infection has been ruled out [20].

Picornaviruses are stable in the environment, which necessitates aggressive disinfection and sanitation methods. Frequent disinfection of enclosures and food bowls should be performed with disinfectants that are proven to kill picornaviruses, e.g., sodium hypochlorite, acetic acid, sodium carbonate, sodium hydroxide, formaldehyde, ethylene oxide, or potassium peroxymonosulfate with sodium chloride [85].

### 6.3. Immunization

Several attempts have been made at developing a vaccine against EMCV, with variable success. To the authors’ knowledge, no commercially available vaccines are currently marketed. As a result, heavy emphasis should be placed on rodent control and appropriate disinfection practices.

#### 6.3.1. Inactivated Vaccines

One of the earliest attempts at creating a vaccine followed shortly after an outbreak in a zoo in New Orleans. In this study, EMCV was inactivated with formaldehyde. Six domestic cats (*Felis catus*), two common brown lemurs (*Eulemur fulvus*), two black-and-white ruffed lemurs (*Varecia variegata*), three black-handed spider monkeys (*Ateles geoffroyi*), three mantled guerezas (*Colobus guereza*), and two tufted capuchin monkeys (*Cebus apella*) received 1–2 mL vaccine IM. One dromedary (*Camelus dromedarius*) received 5 mL. All animals received a second IM injection 3–4 weeks later. Five out of six cats, the spider monkeys, and the black-and-white ruffed lemurs developed titers of 1:20–1:80 after booster vaccination. Unfortunately, no antibody titers were measured in the other animals. No adverse reactions were observed following vaccination [69].

An inactivated EMCV vaccine, derived from an isolate during an outbreak among elephants in Kruger National Park, was tested in mice, pigs, and elephants. This vaccine was prepared with a double emulsion Montanide ISA 206 oil adjuvant and contains 16 mg/mL EMCV particles. This vaccine conferred protection against live virus challenge in mice, pigs, and elephants. Piglets (*n* = 4) received an IM administration of 1 mL vaccine and were subsequently challenged 25 days post-vaccination. They developed robust antibody responses, and no clinical signs or mortalities were observed. Elephant calves (*n* = 12) received 5 mL of the vaccine, of which six animals were challenged approximately two months after vaccination. No adverse effects were observed, and no clinical signs were reported following virus challenge in vaccinated animals. Two unvaccinated elephants died, and one showed malaise and ECG abnormalities. One unvaccinated animal developed subclinical infection, with lesions compatible with EMCV infection upon pathological examination. Virus could not be isolated from the feces of the vaccinated animals or from the fatal cases [29]. Three free-ranging, adult elephant bulls were vaccinated. In these animals, titers were demonstrated to persist for at least 12 months [29].

A similar vaccine preparation using an Australian strain was used in Barbary sheep (*Ammotragus lervia*) (*n* = 34), Indian antelopes (*Antilope cervicapra*) (*n* = 30), Eastern wallaroos (*Macropus robustus*) (*n* = 14), and chimpanzees (*n* = 8). Following a single 2 mL IM vaccination, significant differences in antibody responses were noted compared to animals receiving a booster immunization after one month. Moreover, in some individuals, particularly antelopes, antibody titers declined rapidly between 3 and 12 months after vaccination [86]. Although no live virus challenge was performed, the antibody titers observed are similar to those described in the South African study and are likely protective [29,86].

A different vaccine has been derived from an inactivated isolate cultured from an elephant during an outbreak in a zoo in Florida. This vaccine was grown in baby hamster kidney cells, inactivated with b-propiolactone, and prepared as an oil emulsion vaccine. Fourfold increases in titers were observed in 74% (34/45) of rhesus macaques kept in a primate research center four months following a single 1 mL IM injection. At 18 months post-vaccination, this titer persisted in 62% (28/45) of animals. Unfortunately, no virus challenge was performed to validate the protective efficacy of these titers [87]. Interestingly, an inactivated vaccine derived from a porcine field strain did not produce titers in any of the macaques (*n* = 51) in the same study. This vaccine was prepared with the same techniques but was inactivated with binary ethyleneamine and contained aluminum hydroxide as an adjuvant [87].

Although some inactivated vaccines are demonstrated to confer protection against EMCV, it has been reported that high antibody titers do not necessarily protect against disease. A mandrill kept in a zoological institution survived a first natural infection but succumbed to a second natural EMCV infection three years after the initial infection, in spite of having measurable antibody titers [27]. A fatal EMCV infection occurred in a captive capybara in spite of annual IM immunization using an inactivated vaccine. Four weeks following vaccination, a virus neutralization titer of 320 was measured, which is considered to be protective in pigs. Two conspecifics that demonstrated no signs of disease had titers of 640 and 5012, but it is unknown whether these animals were exposed to the virus [43]. It must be considered that the protective efficacy of antibodies may depend on the virus lineage and/or serotype involved. Further research should be conducted to determine the extent to which cross-protection occurs between different EMCV lineages and serotypes following vaccination and natural exposure.

#### 6.3.2. Live Attenuated Vaccines

Vaccination with an attenuated, short poly-(C) mengovirus has been demonstrated to confer protection against wildtype EMCV challenge in macaques, baboons, and pigs [80]. This vaccine strategy has been applied to a wide range of species in a zoological setting, including various species of NHPs, tapirs, camelids, bovids, suids, and porcupines. In this study, all animals were injected IM with 5.0 × 10^6^ plaque-forming units of culture supernatant. Blood was collected before and 21 days after immunization [88]. Seroconversion, as indicated by a greater than four fold increase in serum titer, was reported in only some individuals: a single dromedary (1/7), a Baird’s tapir (*Tapirus bairdii*) (1/4), a cape porcupine (*Hystrix africaeaustralis*) (1/2), gerenuks (*Litocranius walleri*) (3/8), collared mangabeys (*Cercocebus torquatus*) (2/6), Angolan colobus monkeys (*Colobus angolensis*) (5/11), and a Diana monkey (*Cercopithecus diana*) (1/2). Only in talapoins (*Miopithecus talapoin*) did all animals seroconvert (6/6). Although this vaccine appears to be most effective in NHPs, no seroconversion was observed in vaccinated common marmosets (*n* = 3), mantled guerezas (*n* = 4), siamangs (*Hylobates syndactylus*) (*n* = 2), or any of the lemurs (*n* = 12) [88]. Although no virus challenge was performed, the authors considered serum neutralizing antibody titers exceeding 1:16 to be protective, based on trials in baboons, pigs, and mice [80].

## 7. Conclusions

EMCV is a zoonotic virus that poses a significant threat to both domesticated and non-domesticated animals worldwide. In domestic species, there may be large economic consequences of EMCV outbreaks. Epizootic events may occur in a wide range of species and cause significant morbidity and mortality. Despite its impact, EMCV remains relatively unknown. Clinicians should consider EMCV as a differential diagnosis when faced with cases of peracute death or unexplained acute heart failure in zoological collections. Rapid diagnostic methods, such as RT-PCR, are available and can facilitate timely diagnosis. However, increasing clinician awareness is required to ensure these diagnostic tools are utilized promptly. Early detection and intervention may improve the chances of successful treatment.

Due to the rapid disease progression, often fatal nature of EMCV-induced myocarditis, and difficulties associated with its treatment, this review underscores the importance of preventative measures. Biosecurity protocols should be implemented to minimize rodent contamination of feed and water supplies. Both inactivated and live attenuated vaccines have demonstrated varying degrees of efficacy across different species but are not commercially available. Moreover, the reported vaccines have often not been validated under laboratory conditions. Future investigations should prioritize enhancing vaccine immunogenicity and evaluating vaccine efficacy in a wide range of species under controlled conditions.

## Data Availability

Data are available upon reasonable request.

## Appendix A

**Table A1:** An overview of clinical signs as reported in referred literature associated with EMCV cases in zoological institutions.

							General				Cardiovascular System			Respiratory System			Nervous System				
Species’ Order	Species’ Family	Species (Latin Name)	Number of Animals (*n*)	Sex (M/F/NA)	Age Avg. (Years) (Range)	Clinical Signs (Yes/No/NA)	Anorexia or Hyporexia (Yes/No/NA)	Lethargy (Yes/No/NA)	Recumbency (Yes/No/NA)	Colic (Yes/No/NA)	Unspecified Signs of Cardiac Failure (Yes/No/NA)	Bradyarrythmia (Yes/No/NA)	ECG Abnormalities (Yes/No/NA)	Tachypnea (Yes/No/NA)	Dyspnea (Yes/No/NA)	Nasal Discharge (Yes/No/NA)	Ataxia (Yes/No/NA)	Hemiparesis (Yes/No/NA)	Tremors (Yes/No/NA)	Death (Yes/No/NA)	Reference
Diprodontia	Macropodidae	Goodfellow’s tree kangaroo (*Dendrolagus goodfellowi*)	2	1/1/0	2 (2)	0.0% (0/2/0)	NA (0/0/2)	NA (0/0/2)	NA (0/0/2)	NA (0/0/2)	NA (0/0/2)	NA (0/0/2)	NA (0/0/2)	NA (0/0/2)	NA (0/0/2)	NA (0/0/2)	NA (0/0/2)	NA (0/0/2)	NA (0/0/2)	100.0% (2/0/0)	[27]
Artiodactyla	Bovidae	Oryx-addax antelope hybrid (*Oryx* sp. × *Addax nasomaculatus*)	1	1/0/0	0.2	100.0% (1/0/0)	100.0% (1/0/0)	0.0% (0/1/0)	0.0% (0/1/0)	0.0% (0/1/0)	0.0% (0/1/0)	0.0% (0/1/0)	0.0% (0/1/0)	0.0% (0/1/0)	0.0% (0/1/0)	0.0% (0/1/0)	0.0% (0/1/0)	0.0% (0/1/0)	0.0% (0/1/0)	100.0% (1/0/0)	[20]
	Camelidae	Alpaca (*Lama pacos*)	6	3/3/0	8.2 (3–13)	100.0% (6/0/0)	16.7% (1/5/0)	33.3% (2/4/0)	83.3% (5/1/0)	83.3% (5/1/0)	0.0% (0/6/0)	0.0% (0/6/0)	0.0% (0/6/0)	0.0% (0/6/0)	0.0% (0/6/0)	0.0% (0/6/0)	33.3% (2/4/0)	0.0% (0/6/0)	0.0% (0/6/0)	100.0% (6/0/0)	[44]
	Hippopotamidae	Pygmy hippopotamus (*Choeropsis liberiensis*)	1	0/1/0	12	100.0% (1/0/0)	100.0% (1/0/0)	100.0% (1/0/0)	0.0% (0/1/0)	0.0% (0/1/0)	0.0% (0/1/0)	0.0% (0/1/0)	0.0% (0/1/0)	0.0% (0/1/0)	0.0% (0/1/0)	0.0% (0/1/0)	0.0% (0/1/0)	0.0% (0/1/0)	0.0% (0/1/0)	100.0% (1/0/0)	[27]
	Suidae	Boar (*Sus scrofa*)	38	0/0/38	NA	NA (0/0/38) *	NA (0/0/38) *	NA (0/0/38)	NA (0/0/38)	NA (0/0/38)	NA (0/0/38)	NA (0/0/38)	NA (0/0/38)	NA (0/0/38) *	NA (0/0/38)	NA (0/0/38)	NA (0/0/38) *	NA (0/0/38)	NA (0/0/38)	100.0% (38/0/0)	[58]
Carnivora	Felidae	South China tiger (*Panthera tigris amoyensis*)	3	1/2/0	12.7 (9–16)	66.6% (2/1/0)	100.0% (2/0/1)	50.0% (1/1/1)	0.0% (0/2/1)	0.0% (0/2/1)	0.0% (0/2/1)	0.0% (0/2/1)	0.0% (0/2/1)	0.0% (0/2/1)	0.0% (0/2/1)	0.0% (0/2/1)	0.0% (0/2/1)	0.0% (0/2/1)	0.0% (0/2/1)	100.0% (3/0/0)	[72]
Perissodactyla	Tapiridae	Malayan tapir (*Tapirus indicus*)	1	0/1/0	0.4	0.0% (0/1/0)	NA (0/0/1)	NA (0/0/1)	NA (0/0/1)	NA (0/0/1)	NA (0/0/1)	NA (0/0/1)	NA (0/0/1)	NA (0/0/1)	NA (0/0/1)	NA (0/0/1)	NA (0/0/1)	NA (0/0/1)	NA (0/0/1)	100.0% (1/0/0)	[53]
Primates	Callitrichidae	Common marmoset (*Callithrix jacchus*)	1	1/0/0	NA	0.0% (0/1/0)	NA (0/0/1)	NA (0/0/1)	NA (0/0/1)	NA (0/0/1)	NA (0/0/1)	NA (0/0/1)	NA (0/0/1)	NA (0/0/1)	NA (0/0/1)	NA (0/0/1)	NA (0/0/1)	NA (0/0/1)	NA (0/0/1)	100.0% (1/0/0)	[17]
	Cebidae	Squirrel monkey (*Saimiri sciureus*)	1	0/1/0	>5	0.0% (0/1/0)	NA (0/0/1)	NA (0/0/1)	NA (0/0/1)	NA (0/0/1)	NA (0/0/1)	NA (0/0/1)	NA (0/0/1)	NA (0/0/1)	NA (0/0/1)	NA (0/0/1)	NA (0/0/1)	NA (0/0/1)	NA (0/0/1)	100.0% (1/0/0)	[27]
	Cercopithecidae	Barbary macaque (*Macaca sylvanus*)	7	1/0/6	NA	NA (0/3/4) *	NA (0/0/7)	NA (0/0/7) *	NA (0/0/7)	NA (0/0/7)	NA (0/0/7)	NA (0/0/7)	NA (0/0/7)	NA (0/0/7)	NA (0/0/7)	NA (0/0/7)	NA (0/0/7)	NA (0/0/7)	NA (0/0/7)	100.0% (7/0/0)	[17,28]
		De Brazza’s monkey (*Cercopithecus neglectus*)	1	1/0/0	NA	0.0% (0/1/0)	NA (0/0/1)	NA (0/0/1)	NA (0/0/1)	NA (0/0/1)	NA (0/0/1)	NA (0/0/1)	NA (0/0/1)	NA (0/0/1)	NA (0/0/1)	NA (0/0/1)	NA (0/0/1)	NA (0/0/1)	NA (0/0/1)	100.0% (1/0/0)	[20]
		Grivet (*Cercopithecus aethiops*)	3	0/0/3	NA	0.0% (0/3/0)	NA (0/0/3)	NA (0/0/3)	NA (0/0/3)	NA (0/0/3)	NA (0/0/3)	NA (0/0/3)	NA (0/0/3)	NA (0/0/3)	NA (0/0/3)	NA (0/0/3)	NA (0/0/3)	NA (0/0/3)	NA (0/0/3)	0.0% (0/3/0)	[24]
		Hamadryas baboon (*Papio hamadryas*)	56	0/0/56	NA	NA (0/0/56)	NA (0/0/56)	NA (0/0/56)	NA (0/0/56)	NA (0/0/56)	NA (0/0/56)	NA (0/0/56)	NA (0/0/56)	NA (0/0/56)	NA (0/0/56)	NA (0/0/56)	NA (0/0/56)	NA (0/0/56)	NA (0/0/56)	100.0% (56/0/0)	[24,30]
		Mandrill (*Mandrillus sphinx*)	3	2/1/0	7 (3–10)	0.0% (0/3/0)	NA (0/0/3)	NA (0/0/3)	NA (0/0/3)	NA (0/0/3)	NA (0/0/3)	NA (0/0/3)	NA (0/0/3)	NA (0/0/3)	NA (0/0/3)	NA (0/0/3)	NA (0/0/3)	NA (0/0/3)	NA (0/0/3)	100.0% (3/0/0)	[27]
		Rhesus macaque (*Macaca mulatta*)	16	4/2/10	NA	50.0% (3/3/10) *	0.0% (0/3/13)	33.3% (1/2/13)	0.0% (0/3/13)	0.0% (0/3/13)	0.0% (0/3/13)	0.0% (0/3/13)	0.0% (0/3/13)	66.6% (2/1/13)	66.6% (2/1/13)	0.0% (0/3/13)	0.0% (0/3/13)	0.0% (0/3/13)	0.0% (0/3/13)	100.0% (16/0/0)	[24,26]
	Hominidae	Bonobo (*Pan paniscus*)	2	0/0/2	3.5 (2–5)	100.0% (2/0/0)	0.0% (0/2/0)	0.0% (0/2/0)	0.0% (0/2/0)	0.0% (0/2/0)	0.0% (0/2/0)	0.0% (0/2/0)	0.0% (0/2/0)	100.0% (2/0/0)	100.0% (2/0/0)	0.0% (0/2/0)	0.0% (0/2/0)	0.0% (0/2/0)	0.0% (0/2/0)	100.0% (2/0/0)	[23]
		Chimpanzee (Pan troglodytes)	5	3/2/0	8.4 (2–19)	3/2/0	0.0% (0/3/2)	66.6% (2/1/2)	0.0% (0/3/2)	0.0% (0/3/2)	33.3% (1/2/2)	0.0% (0/3/2)	0.0% (0/3/2)	0.0% (0/3/2)	33.3% (1/2/2)	0.0% (0/3/2)	33.3% (1/2/2)	0.0% (0/3/2)	0.0% (0/3/2)	100.0% (5/0/0)	[20,27]
		Orangutan (*Pongo* sp.)	4	2/2/0	NA	NA (0/0/4)	NA (0/0/4)	NA (0/0/4)	NA (0/0/4)	NA (0/0/4)	NA (0/0/4)	NA (0/0/4)	NA (0/0/4)	NA (0/0/4)	NA (0/0/4)	NA (0/0/4)	NA (0/0/4)	NA (0/0/4)	NA (0/0/4)	100.0% (4/0/0)	[31]
		Sumatran orangutan (*Pongo abelii*)	1	1/0/0	23	100.0% (1/0/0)	100.0% (1/0/0)	0.0% (0/1/0)	0.0% (0/1/0)	0.0% (0/1/0)	0.0% (0/1/0)	0.0% (0/1/0)	0.0% (0/1/0)	0.0% (0/1/0)	0.0% (0/1/0)	0.0% (0/1/0)	0.0% (0/1/0)	0.0% (0/1/0)	0.0% (0/1/0)	100.0% (1/0/0)	[18]
	Lemuridae	Black lemur (*Eulemur macaco*)	1	1/0/0	NA	100.0% (1/0/0)	100.0% (1/0/0)	100.0% (1/0/0)	0.0% (0/1/0)	0.0% (0/1/0)	0.0% (0/1/0)	0.0% (0/1/0)	0.0% (0/1/0)	0.0% (0/1/0)	0.0% (0/1/0)	0.0% (0/1/0)	0.0% (0/1/0)	0.0% (0/1/0)	0.0% (0/1/0)	100.0% (1/0/0)	[17]
		Red-ruffed (*Varecia variegata rubra*)	6	2/2/2	NA	16.7% (1/5/0)	0.0% (0/1/5)	100.0% (1/0/5)	0.0% (0/1/5)	0.0% (0/1/5)	0.0% (0/1/5)	0.0% (0/1/5)	0.0% (0/1/5)	0.0% (0/1/5)	0.0% (0/1/5)	0.0% (0/1/5)	0.0% (0/1/5)	0.0% (0/1/5)	0.0% (0/1/5)	100.0% (6/0/0)	[17]
		Ring-tailed lemur (*Lemur catta*)	3	2/0/1	NA	33.3% (1/2/0)	0.0% (0/1/2)	0.0% (0/1/2)	0.0% (0/1/2)	0.0% (0/1/2)	0.0% (0/1/2)	0.0% (0/1/2)	0.0% (0/1/2)	0.0% (0/1/2)	0.0% (0/1/2)	0.0% (0/1/2)	100.0% (1/0/2)	0.0% (0/1/2)	0.0% (0/1/2)	100.0% (3/0/0)	[17]
		Ring-tailed lemur (*Lemur catta*)	1	1/0/0	7	0.0% (0/1/0)	NA (0/0/1)	NA (0/0/1)	NA (0/0/1)	NA (0/0/1)	NA (0/0/1)	NA (0/0/1)	NA (0/0/1)	NA (0/0/1)	NA (0/0/1)	NA (0/0/1)	NA (0/0/1)	NA (0/0/1)	NA (0/0/1)	100.0% (1/0/0)	[27]
		White-fronted lemur (*Eulemur albifrons*)	2	2/0/0	NA	50.0% (1/1/0)	0.0% (0/1/1)	100.0% (1/0/1)	0.0% (0/1/1)	0.0% (0/1/1)	0.0% (0/1/1)	0.0% (0/1/1)	0.0% (0/1/1)	0.0% (0/1/1)	0.0% (0/1/1)	0.0% (0/1/1)	0.0% (0/1/1)	0.0% (0/1/1)	0.0% (0/1/1)	100.0% (2/0/0)	[17]
Proboscidea	Elephantidae	African elephant (*Loxodonta africana*)	20	0/6/14	NA	61.5% (8/5/7)	0.0% (0/8/12)	25.0% (2/6/12)	12.5% (1/7/12)	0.0% (0/8/12)	0.0% (0/8/12)	0.0% (0/8/12)	12.5% (1/7/12)	0.0% (0/8/12)	12.5% (1/7/12)	12.5% (1/7/12)	12.5% (1/7/12)	0.0% (0/8/12)	12.5% (1/7/12)	80.0% (15/4/1)+~	[20,21,25,29,71]
		Asian elephant (*Elephas maximus*)	1	1/0/0	9	100.0% (1/0/0)	0.0% (0/1/0)	0.0% (0/1/0)	0.0% (0/1/0)	100.0% (1/0/0)	0.0% (0/1/0)	0.0% (0/1/0)	0.0% (0/1/0)	0.0% (0/1/0)	0.0% (0/1/0)	0.0% (0/1/0)	0.0% (0/1/0)	0.0% (0/1/0)	0.0% (0/1/0)	100.0% (1/0/0)	[20]
Rodentia	Caviidae	Capybara (*Hydrochoerus hydrochaeris*)	2	1/0/1	NA	100.0% (2/0/0)	0.0% (0/2/0)	100.0% (2/0/0)	0.0% (0/2/0)	0.0% (0/2/0)	0.0% (0/2/0)	50.0% (1/1/0)	50.0% (1/1/0)	0.0% (0/2/0)	0.0% (0/2/0)	50.0% (1/1/0)	50.0% (1/1/0)	50.0% (1/1/0)	0.0% (0/2/0)	100.0% (2/0/0)	[43,69]
	Hystricidae	Crested porcupine (*Hystrix cristata*)	24	0/0/24	NA	NA (0/0/24)	NA (0/0/24)	NA (0/0/24)	NA (0/0/24)	NA (0/0/24)	NA (0/0/24)	NA (0/0/24)	NA (0/0/24)	NA (0/0/24)	NA (0/0/24)	NA (0/0/24)	NA (0/0/24)	NA (0/0/24)	NA (0/0/24)	100.0% (24/0/0)	[28]
		Total	212	31/24/157		69.2% (72/32/108)	20.6% (7/27/178)	41.2% (14/20/178)	17.6% 6/28/178)	17.6% (6/28/178)	2.9% (1/33/178)	2.9% (1/33/178)	2.9% (1/33/178)	5.9% (2/32/178)	17.6% 6/28/178)	5.9% (2/32/178)	17.6% 6/28/178)	2.9% (1/33/178)	2.9% (1/33/178)	96.7% (204/7/1)	

* Although this clinical sign was reported, the number of affected animals is not available. + Four animals died not of EMCV but were sacrificed as part of an experiment. ~ ‘NA’ animals were euthanized due to the severity of clinical signs. M = male; F = Female; NA is used to denote the absence of data for a parameter. Cell background color is used to indicate that the clinical sign is reported in this species.

**Table A2:** An overview of gross postmortem lesions as reported in referred literature associated with EMCV cases in zoological institutions.

						General		Cardiovascular System						Pulmonary System					Nervous System	
Species’ Order	Species’ Family	Species (Latin Name)	Number of Animals (*n*)	Sex (M/F/NA)	Age Avg. (Years) (Range)	Free Fluid in the Thoracic Cavity (Yes/No/NA)	Free Fluid in the Abdominal Cavity (Yes/No/NA)	Pericardial Fluid (Yes/No/NA)	Pale Foci in the Myocardium (Yes/No/NA)	Cardiomegaly (Yes/No/NA)	Petechial or Ecchymotic Hemorrhages on the Heart (Yes/No/NA)		Soft Heart Consistency (Yes/No/NA)	Pulmonary Edema (Yes/No/NA)	Pulmonary Congestion (Yes/No/NA)	Foam in Trachea (Yes/No/NA)	Pulmonary Emphysema (Yes/No/NA)	Dark Mottling on the Lung (Yes/No/NA)	Meningeal Congestion (Yes/No/NA)	Reference
Diprodontia	Macropodidae	Goodfellow’s tree kangaroo (*Dendrolagus goodfellowi*)	2	1/1/0	2 (2)	0.0% (0/2/0)	50.0% (1/1/0)	0.0% (0/2/0)	50.0% (1/1/0)	0.0% (0/2/0)	0.0% (0/2/0)		0.0% (0/2/0)	0.0% (0/2/0)	100.0% (2/0/0)	0.0% (0/2/0)	0.0% (0/2/0)	0.0% (0/2/0)	0.0% (0/2/0)	[27]
Artiodactyla	Bovidae	Oryx-addax antelope hybrid (*Oryx* sp. × *Addax nasomaculatus*)	1	1/0/0	0.2	0.0% (0/1/0)	0.0% (0/1/0)	0.0% (0/1/0)	100.0% (1/0/0)	0.0% (0/1/0)	0.0% (0/1/0)		0.0% (0/1/0)	100.0% (1/0/0)	0.0% (0/1/0)	100.0% (1/0/0)	0.0% (0/1/0)	0.0% (0/1/0)	0.0% (0/1/0)	[20]
	Camelidae	Alpaca (*Lama pacos*)	6	3/3/0	8.2 (3–13)	100.0% (6/0/0)	100.0% (6/0/0)	100.0% (6/0/0)	83.3% (5/1/0)	0.0% (0/6/0)	0.0% (0/6/0)		0.0% (0/6/0)	33.3% (2/4/0)	50.0% (3/3/0)	33.3% (2/4/0)	0.0% (0/6/0)	0.0% (0/6/0)	0.0% (0/6/0)	[44]
	Hippopotamidae	Pygmy hippopotamus (*Choeropsis liberiensis*)	1	0/1/0	12	0.0% (0/1/0)	100.0% (1/0/0)	100.0% (1/0/0)	100.0% (1/0/0)	0.0% (0/1/0)	0.0% (0/1/0)		0.0% (0/1/0)	0.0% (0/1/0)	100.0% (1/0/0)	0.0% (0/1/0)	0.0% (0/1/0)	0.0% (0/1/0)	0.0% (0/1/0)	[27]
	Suidae	Boar (*Sus scrofa*)	38	0/0/38	NA	0.0% (0/38/0)	0.0% (0/38/0)	NA (0/0/38) *	0.0% (0/38/0)	0.0% (0/38/0)	0.0% (0/38/0)		NA (0/0/38) *	0.0% (0/38/0)	0.0% (0/38/0)	0.0% (0/38/0)	0.0% (0/38/0)	0.0% (0/38/0)	0.0% (0/38/0)	[58]
Carnivora	Felidae	South China tiger (*Panthera tigris amoyensis*)	3	1/2/0	12.7 (9–16)	33.3% (1/2/0)	33.3% (1/2/0)	33.3% (1/2/0)	0.0% (0/3/0)	33.3% (1/2/0)	33.3% (1/2/0)		0.0% (0/3/0)	33.3% (1/2/0)	0.0% (0/3/0)	0.0% (0/3/0)	33.3% (1/2/0)	0.0% (0/3/0)	33.3% (1/2/0)	[72]
Perissodactyla	Tapiridae	Malayan tapir (*Tapirus indicus*)	1	0/1/0	0.4	0.0% (0/1/0)	100.0% (1/0/0)	0.0% (0/1/0)	100.0% (1/0/0)	0.0% (0/1/0)	0.0% (0/1/0)		0.0% (0/1/0)	0.0% (0/1/0)	100.0% (1/0/0)	100.0% (1/0/0)	0.0% (0/1/0)	0.0% (0/1/0)	0.0% (0/1/0)	[53]
Primates	Callitrichidae	Common marmoset (*Callithrix jacchus*)	1	1/0/0	NA	0.0% (0/1/0)	100.0% (1/0/0)	0.0% (0/1/0)	100.0% (1/0/0)	100.0% (1/0/0)	0.0% (0/1/0)		0.0% (0/1/0)	100.0% (1/0/0)	0.0% (0/1/0)	0.0% (0/1/0)	0.0% (0/1/0)	0.0% (0/1/0)	0.0% (0/1/0)	[17]
	Cebidae	Squirrel monkey (*Saimiri sciureus*)	1	0/1/0	>5	0.0% (0/1/0)	0.0% (0/1/0)	0.0% (0/1/0)	0.0% (0/1/0)	0.0% (0/1/0)	0.0% (0/1/0)		0.0% (0/1/0)	100.0% (1/0/0)	0.0% (0/1/0)	0.0% (0/1/0)	0.0% (0/1/0)	0.0% (0/1/0)	0.0% (0/1/0)	[27]
	Cercopithecidae	Barbary macaque (*Macaca sylvanus*)	7	1/0/6	NA	57.1% (4/3/0)	0.0% (0/7/0)	57.1% (4/3/0)	0.0% (0/7/0)	0.0% (0/7/0)	0.0% (0/7/0)		0.0% (0/7/0)	71.4% (5/2/0)	57.1% (4/3/0)	0.0% (0/7/0)	0.0% (0/7/0)	0.0% (0/7/0)	57.1% (4/3/0)	[17,28]
		De Brazza’s monkey (*Cercopithecus neglectus*)	1	1/0/0	NA	NA (0/0/1)	NA (0/0/1)	NA (0/0/1)	NA (0/0/1)	NA (0/0/1)	NA (0/0/1)		NA (0/0/1)	NA (0/0/1)	NA (0/0/1)	NA (0/0/1)	NA (0/0/1)	NA (0/0/1)	NA (0/0/1)	[20]
		Grivet (*Cercopithecus aethiops*)	3	0/0/3	NA	100.0% (3/0/0)	0.0% (0/3/0)	100.0% (3/0/0)	NA (0/0/3) *	0.0% (0/3/0)	0.0% (0/3/0)		0.0% (0/3/0)	100.0% (3/0/0)	100.0% (3/0/0)	0.0% (0/3/0)	0.0% (0/3/0)	0.0% (0/3/0)	0.0% (0/3/0)	[24]
		Hamadryas baboon (*Papio hamadryas*)	56	0/0/56	NA	51.8% (29/27/0)	0.0% (0/56/0)	100.0% (29/0/27) *	NA (0/27/29) *	NA (0/29/27) *	0.0% (0/56/0)		0.0% (0/56/0)	51.8% (29/27/0)	51.8% (29/27/0)	0.0% (0/56/0)	0.0% (0/56/0)	0.0% (0/56/0)	0.0% (0/56/0)	[24,30]
		Mandrill (*Mandrillus sphinx*)	3	2/1/0	7 (3–10)	0.0% (0/3/0)	33.3% (1/2/0)	33.3% (1/2/0)	66.6% (2/1/0)	0.0% (0/3/0)	33.3% (1/2/0)		0.0% (0/3/0)	0.0% (0/3/0)	33.3% (1/2/0)	0.0% (0/3/0)	0.0% (0/3/0)	0.0% (0/3/0)	0.0% (0/3/0)	[27]
		Rhesus macaque (*Macaca mulatta*)	16	4/2/10	NA	87.5% (14/2/0)	0.0% (0/16/0)	68.8% (11/5/0)	50.0% (3/3/10) *	0.0% (0/16/0)	0.0% (0/16/0)		0.0% (0/16/0)	62.5% (10/6/0)	100.0% (16/0/0)	25.0% (4/12/0)	0.0% (0/16/0)	0.0% (0/16/0)	0.0% (0/16/0)	[24,26]
	Hominidae	Bonobo (*Pan paniscus*)	2	0/0/2	3.5 (2–5)	0.0% (0/2/0)	0.0% (0/2/0)	0.0% (0/2/0)	0.0% (0/2/0)	0.0% (0/2/0)	0.0% (0/2/0)		0.0% (0/2/0)	0.0% (0/2/0)	100.0% (2/0/0)	0.0% (0/2/0)	0.0% (0/2/0)	0.0% (0/2/0)	0.0% (0/2/0)	[23]
		Chimpanzee (Pan troglodytes)	5	3/2/0	8.4 (2–19)	0.0% (0/5/0)	0.0% (0/5/0)	0.0% (0/5/0)	100/0% (5/0/0)	0.0% (0/5/0)	20.0% (1/4/0)		0.0% (0/5/0)	40.0% (2/3/0)	0.0% (0/5/0)	0.0% (0/5/0)	0.0% (0/5/0)	0.0% (0/5/0)	0.0% (0/5/0)	[20,27]
		Orangutan (*Pongo* sp.)	4	2/2/0	NA	NA (0/0/4) *	NA (0/0/4) *	NA (0/0/4) *	0.0% (0/4/0)	0.0% (0/4/0)	0.0% (0/4/0)		0.0% (0/4/0)	NA (0/0/4) *	NA (0/0/4) *	0.0% (0/4/0)	0.0% (0/4/0)	0.0% (0/4/0)	0.0% (0/4/0)	[31]
		Sumatran orangutan (*Pongo abelii*)	1	1/0/0	23	0.0% (0/1/0)	100.0% (1/0/0)	100.0% (1/0/0)	100.0% (1/0/0)	100.0% (1/0/0)	0.0% (0/1/0)		0.0% (0/1/0)	100.0% (1/0/0)	0.0% (0/1/0)	100.0% (1/0/0)	0.0% (0/1/0)	100.0% (1/0/0)	0.0% (0/1/0)	[18]
	Lemuridae	Black lemur (*Eulemur macaco*)	1	1/0/0	NA	0.0% (0/1/0)	0.0% (0/1/0)	0.0% (0/1/0)	0.0% (0/1/0)	0.0% (0/1/0)	0.0% (0/1/0)		0.0% (0/1/0)	100.0% (1/0/0)	0.0% (0/1/0)	0.0% (0/1/0)	0.0% (0/1/0)	0.0% (0/1/0)	0.0% (0/1/0)	[17]
		Red-ruffed (*Varecia variegata rubra*)	6	2/2/2	NA	16.7% (1/5/0)	0.0% (0/6/0)	0.0% (0/6/0)	100.0% (6/0/0)	33.3% (2/4/0)	0.0% (0/6/0)		0.0% (0/6/0)	100.0% (6/0/0)	0.0% (0/6/0)	0.0% (0/6/0)	0.0% (0/6/0)	0.0% (0/6/0)	0.0% (0/6/0)	[17]
		Ring-tailed lemur (*Lemur catta*)	3	2/0/1	NA	33.3% (1/2/0)	0.0% (0/3/0)	0.0% (0/3/0)	33.3% (1/2/0)	33.3% (1/2/0)	66.6% (2/1/0)		0.0% (0/3/0)	66.6% (2/1/0)	0.0% (0/3/0)	0.0% (0/3/0)	66.6% (2/1/0)	0.0% (0/3/0)	66.6% (2/1/0)	[17]
		Ring-tailed lemur (*Lemur catta*)	1	1/0/0	7	0.0% (0/1/0)	0.0% (0/1/0)	0.0% (0/1/0)	100.0% (1/0/0)	0.0% (0/1/0)	0.0% (0/1/0)		0.0% (0/1/0)	100.0% (1/0/0)	0.0% (0/1/0)	0.0% (0/1/0)	0.0% (0/1/0)	0.0% (0/1/0)	0.0% (0/1/0)	[27]
		White-fronted lemur (*Eulemur albifrons*)	2	2/0/0	NA	100.0% (2/0/0)	0.0% (0/2/0)	0.0% (0/2/0)	0.0% (0/2/0)	50.0% (1/1/0)	100.0% (2/0/0)		0.0% (0/2/0)	100.0% (2/0/0)	0.0% (0/2/0)	0.0% (0/2/0)	100.0% (2/0/0)	0.0% (0/2/0)	100.0% (2/0/0)	[17]
Proboscidea	Elephantidae	African elephant (*Loxodonta africana*)	20	0/6/14	NA	35.0% (7/13/0)	45.0% (9/11/0)	45.0% (9/11/0)	76.9% (10/3/7) *	10/0% (2/18/0)	30.8% (4/9/7) *		20.0% (4/16/0)	38.5% (5/8/7) *	23.1% (3/10/7) *	0.0% (0/20/0)	0.0% (0/20/0)	0.0% (0/20/0)	NA (0/13/7) *	[20,21,25,29,71]
		Asian elephant (*Elephas maximus*)	1	1/0/0	9	0.0% (0/1/0)	0.0% (0/1/0)	0.0% (0/1/0)	0.0% (0/1/0)	0.0% (0/1/0)	100.0% (1/0/0)		0.0% (0/1/0)	0.0% (0/1/0)	100.0% (1/0/0)	0.0% (0/1/0)	0.0% (0/1/0)	0.0% (0/1/0)	0.0% (0/1/0)	[20]
Rodentia	Caviidae	Capybara (*Hydrochoerus hydrochaeris*)	2	1/0/1	NA	100.0% (1/0/1)	100.0% (1/0/1)	100.0% (1/0/1)	0.0% (0/1/1)	100.0% (1/0/1)	0.0% (0/1/1)		0.0% (0/1/1)	100.0% (1/0/1)	0.0% (0/1/1)	0.0% (0/1/1)	0.0% (0/1/1)	100.0% (1/0/1)	0.0% (0/1/1)	[43,69]
	Hystricidae	Crested porcupine (*Hystrix cristata*)	24	0/0/24	NA	64.7% (11/6/7)	35.3% (6/11/7)	64.7% (11/6/7)	29.4% (5/12/7)	29.4% (5/12/7)	0.0% (0/17/7)		0.0% (0/17/7)	58.8% (10/7/7)	58.8% (10/7/7)	0.0% (0/17/7)	0.0% (0/17/7)	0.0% (0/17/7)	11.8% (2/15/7)	[28]
		Total	212	31/24/157		40.2% (80/119/13)	14.6% (29/170/13)	58.2% (78/56/78)	28.6% (44/110/58)	8.5% (15/161/36)	6.1% (12/184/16)		2.4% (4/161/47)	43.8% (84/108/20)	39.6% (76/116/20)	4.4% (9/194/9)	2.5% (5/198/9)	1.0% (2/201/9)	5.6% (11/185/16)	
						**Gastrointestinal System**			**Hepatobiliary System**				**Lymphatic System**				**Renal System**			
**Species’ Order**	**Species’ Family**	**Species** **(Latin Name)**	**Number of Animals (*n*)**	**Sex (M/F/NA)**	**Age Avg. (Years)** **(Range)**	**Enteritis** **(Yes/No/NA)**	**Small Intestinal Congestion** **(Yes/No/NA)**	**Hemorrhagic Intestinal Content** **(Yes/No/NA)**	**Hepatic Congestion** **(Yes/No/NA)**	**Hepatomegaly** **(Yes/No/NA)**	**Zonal Liver Pattern** **(Yes/No/NA)**	**Multifocal Hepatic Pale Areas** **(Yes/No/NA)**	**Lymphadenomegaly** **(Yes/No/NA)**	**Congestion of Mesenterial Lymph Nodes** **(Yes/No/NA)**	**Splenomegaly** **(Yes/No/NA)**	**Splenic Congestion** **(Yes/No/NA)**	**Renal Congestion** **(Yes/No/NA)**	**Renal Cortical Pallor** **(Yes/No/NA)**	**Renal Hemorrhage** **(Yes/No/NA)**	**Reference**
Diprodontia	Macropodidae	Goodfellow’s tree kangaroo (*Dendrolagus goodfellowi*)	2	1/1/0	2 (2)	0.0% (0/2/0)	0.0% (0/2/0)	0.0% (0/2/0)	0.0% (0/2/0)	0.0% (0/2/0)	0.0% (0/2/0)	0.0% (0/2/0)	0.0% (0/2/0)	0.0% (0/2/0)	0.0% (0/2/0)	0.0% (0/2/0)	0.0% (0/2/0)	0.0% (0/2/0)	0.0% (0/2/0)	[27]
Artiodactyla	Bovidae	Oryx-addax antelope hybrid (*Oryx* sp. × *Addax nasomaculatus*)	1	1/0/0	0.2	0.0% (0/1/0)	0.0% (0/1/0)	0.0% (0/1/0)	0.0% (0/1/0)	0.0% (0/1/0)	0.0% (0/1/0)	100.0% (1/0/0)	0.0% (0/1/0)	0.0% (0/1/0)	0.0% (0/1/0)	0.0% (0/1/0)	0.0% (0/1/0)	0.0% (0/1/0)	0.0% (0/1/0)	[20]
	Camelidae	Alpaca (*Lama pacos*)	6	3/3/0	8.2 (3–13)	0.0% (0/6/0)	33.3% (2/4/0)	33.3% (2/4/0)	100.0% (6/0/0)	0.0% (0/6/0)	33.3% (2/4/0)	0.0% (0/6/0)	0.0% (0/6/0)	0.0% (0/6/0)	0.0% (0/6/0)	0.0% (0/6/0)	0.0% (0/6/0)	0.0% (0/6/0)	0.0% (0/6/0)	[44]
	Hippopotamidae	Pygmy hippopotamus (*Choeropsis liberiensis*)	1	0/1/0	12	0.0% (0/1/0)	0.0% (0/1/0)	0.0% (0/1/0)	100.0% (1/0/0)	0.0% (0/1/0)	0.0% (0/1/0)	0.0% (0/1/0)	0.0% (0/1/0)	0.0% (0/1/0)	0.0% (0/1/0)	0.0% (0/1/0)	0.0% (0/1/0)	0.0% (0/1/0)	0.0% (0/1/0)	[27]
	Suidae	Boar (*Sus scrofa*)	38	0/0/38	NA	0.0% (0/38/0)	0.0% (0/38/0)	0.0% (0/38/0)	0.0% (0/38/0)	0.0% (0/38/0)	0.0% (0/38/0)	0.0% (0/38/0)	0.0% (0/38/0)	0.0% (0/38/0)	0.0% (0/38/0)	0.0% (0/38/0)	0.0% (0/38/0)	0.0% (0/38/0)	0.0% (0/38/0)	[58]
Carnivora	Felidae	South China tiger (*Panthera tigris amoyensis*)	3	1/2/0	12.7 (9–16)	0.0% (0/3/0)	0.0% (0/3/0)	0.0% (0/3/0)	33.3% (1/2/0)	0.0% (0/3/0)	0.0% (0/3/0)	0.0% (0/3/0)	0.0% (0/3/0)	0.0% (0/3/0)	0.0% (0/3/0)	0.0% (0/3/0)	0.0% (0/3/0)	0.0% (0/3/0)	0.0% (0/3/0)	[72]
Perissodactyla	Tapiridae	Malayan tapir (*Tapirus indicus*)	1	0/1/0	0.4	0.0% (0/1/0)	100.0% (1/0/0)	0.0% (0/1/0)	0.0% (0/1/0)	0.0% (0/1/0)	0.0% (0/1/0)	100.0% (1/0/0)	0.0% (0/1/0)	100.0% (1/0/0)	0.0% (0/1/0)	0.0% (0/1/0)	0.0% (0/1/0)	100.0% (1/0/0)	0.0% (0/1/0)	[53]
Primates	Callitrichidae	Common marmoset (*Callithrix jacchus*)	1	1/0/0	NA	0.0% (0/1/0)	0.0% (0/1/0)	0.0% (0/1/0)	0.0% (0/1/0)	100.0% (1/0/0)	0.0% (0/1/0)	0.0% (0/1/0)	100.0% (1/0/0)	0.0% (0/1/0)	0.0% (0/1/0)	0.0% (0/1/0)	0.0% (0/1/0)	100.0% (1/0/0)	0.0% (0/1/0)	[17]
	Cebidae	Squirrel monkey (*Saimiri sciureus*)	1	0/1/0	>5	0.0% (0/1/0)	0.0% (0/1/0)	0.0% (0/1/0)	100.0% (1/0/0)	0.0% (0/1/0)	0.0% (0/1/0)	0.0% (0/1/0)	0.0% (0/1/0)	0.0% (0/1/0)	0.0% (0/1/0)	100.0% (1/0/0)	0.0% (0/1/0)	0.0% (0/1/0)	0.0% (0/1/0)	[27]
	Cercopithecidae	Barbary macaque (*Macaca sylvanus*)	7	1/0/6	NA	14.3% (1/6/0)	0.0% (0/7/0)	0.0% (0/7/0)	57.1% (4/3/0)	0.0% (0/7/0)	0.0% (0/7/0)	0.0% (0/7/0)	0.0% (0/7/0)	0.0% (0/7/0)	0.0% (0/7/0)	0.0% (0/7/0)	57.1% (4/3/0)	0.0% (0/7/0)	0.0% (0/7/0)	[17,28]
		De Brazza’s monkey (*Cercopithecus neglectus*)	1	1/0/0	NA	NA (0/0/1)	NA (0/0/1)	NA (0/0/1)	NA (0/0/1)	NA (0/0/1)	NA (0/0/1)	NA (0/0/1)	NA (0/0/1)	NA (0/0/1)	NA (0/0/1)	NA (0/0/1)	NA (0/0/1)	NA (0/0/1)	NA (0/0/1)	[20]
		Grivet (*Cercopithecus aethiops*)	3	0/0/3	NA	0.0% (0/3/0)	0.0% (0/3/0)	0.0% (0/3/0)	0.0% (0/3/0)	0.0% (0/3/0)	0.0% (0/3/0)	0.0% (0/3/0)	NA (0/0/3) *	0.0% (0/3/0)	0.0% (0/3/0)	0.0% (0/3/0)	0.0% (0/3/0)	0.0% (0/3/0)	0.0% (0/3/0)	[24]
		Hamadryas baboon (*Papio hamadryas*)	56	0/0/56	NA	0.0% (0/56/0)	0.0% (0/56/0)	0.0% (0/56/0)	0.0% (0/56/0)	0.0% (0/56/0)	0.0% (0/56/0)	0.0% (0/56/0)	NA (0/27/29) *	0.0% (0/56/0)	0.0% (0/56/0)	0.0% (0/56/0)	0.0% (0/56/0)	0.0% (0/56/0)	0.0% (0/56/0)	[24,30]
		Mandrill (*Mandrillus sphinx*)	3	2/1/0	7 (3–10)	0.0% (0/3/0)	0.0% (0/3/0)	0.0% (0/3/0)	33.3% (1/2/0)	0.0% (0/3/0)	0.0% (0/3/0)	0.0% (0/3/0)	0.0% (0/3/0)	0.0% (0/3/0)	33.3% (1/2/0)	0.0% (0/3/0)	0.0% (0/3/0)	0.0% (0/3/0)	0.0% (0/3/0)	[27]
		Rhesus macaque (*Macaca mulatta*)	16	4/2/10	NA	0.0% (0/16/0)	0.0% (0/16/0)	0.0% (0/16/0)	6.3% (1/15/0)	12.5% (2/14/0)	0.0% (0/16/0)	0.0% (0/16/0)	NA (0/6/10) *	0.0% (0/16/0)	0.0% (0/16/0)	0.0% (0/16/0)	0.0% (0/16/0)	0.0% (0/16/0)	0.0% (0/16/0)	[24,26]
	Hominidae	Bonobo (*Pan paniscus*)	2	0/0/2	3.5 (2–5)	0.0% (0/2/0)	0.0% (0/2/0)	0.0% (0/2/0)	0.0% (0/2/0)	0.0% (0/2/0)	0.0% (0/2/0)	0.0% (0/2/0)	0.0% (0/2/0)	0.0% (0/2/0)	0.0% (0/2/0)	0.0% (0/2/0)	0.0% (0/2/0)	0.0% (0/2/0)	0.0% (0/2/0)	[23]
		Chimpanzee (Pan troglodytes)	5	3/2/0	8.4 (2–19)	0.0% (0/5/0)	0.0% (0/5/0)	0.0% (0/5/0)	0.0% (0/5/0)	40.0% (2/3/0)	0.0% (0/5/0)	0.0% (0/5/0)	0.0% (0/5/0)	0.0% (0/5/0)	0.0% (0/5/0)	0.0% (0/5/0)	0.0% (0/5/0)	0.0% (0/5/0)	0.0% (0/5/0)	[20,27]
		Orangutan (*Pongo* sp.)	4	2/2/0	NA	0.0% (0/4/0)	0.0% (0/4/0)	0.0% (0/4/0)	0.0% (0/4/0)	0.0% (0/4/0)	0.0% (0/4/0)	0.0% (0/4/0)	0.0% (0/4/0)	0.0% (0/4/0)	0.0% (0/4/0)	0.0% (0/4/0)	0.0% (0/4/0)	0.0% (0/4/0)	0.0% (0/4/0)	[31]
		Sumatran orangutan (*Pongo abelii*)	1	1/0/0	23	0.0% (0/1/0)	0.0% (0/1/0)	0.0% (0/1/0)	100.0% (1/0/0)	100.0% (1/0/0)	0.0% (0/1/0)	0.0% (0/1/0)	0.0% (0/1/0)	0.0% (0/1/0)	100.0% (1/0/0)	100.0% (1/0/0)	0.0% (0/1/0)	0.0% (0/1/0)	0.0% (0/1/0)	[18]
	Lemuridae	Black lemur (*Eulemur macaco*)	1	1/0/0	NA	0.0% (0/1/0)	0.0% (0/1/0)	0.0% (0/1/0)	0.0% (0/1/0)	0.0% (0/1/0)	0.0% (0/1/0)	0.0% (0/1/0)	0.0% (0/1/0)	0.0% (0/1/0)	0.0% (0/1/0)	0.0% (0/1/0)	0.0% (0/1/0)	0.0% (0/1/0)	0.0% (0/1/0)	[17]
		Red-ruffed (*Varecia variegata rubra*)	6	2/2/2	NA	0.0% (0/6/0)	0.0% (0/6/0)	0.0% (0/6/0)	0.0% (0/6/0)	0.0% (0/6/0)	0.0% (0/6/0)	0.0% (0/6/0)	0.0% (0/6/0)	0.0% (0/6/0)	0.0% (0/6/0)	0.0% (0/6/0)	0.0% (0/6/0)	0.0% (0/6/0)	0.0% (0/6/0)	[17]
		Ring-tailed lemur (*Lemur catta*)	3	2/0/1	NA	33.3% (1/2/0)	0.0% (0/3/0)	0.0% (0/3/0)	33.3% (1/2/0)	0.0% (0/3/0)	0.0% (0/3/0)	0.0% (0/3/0)	0.0% (0/3/0)	0.0% (0/3/0)	0.0% (0/3/0)	0.0% (0/3/0)	0.0% (0/3/0)	0.0% (0/3/0)	0.0% (0/3/0)	[17]
		Ring-tailed lemur (*Lemur catta*)	1	1/0/0	7	0.0% (0/1/0)	0.0% (0/1/0)	0.0% (0/1/0)	0.0% (0/1/0)	0.0% (0/1/0)	0.0% (0/1/0)	0.0% (0/1/0)	0.0% (0/1/0)	0.0% (0/1/0)	0.0% (0/1/0)	0.0% (0/1/0)	0.0% (0/1/0)	0.0% (0/1/0)	0.0% (0/1/0)	[27]
		White-fronted lemur (*Eulemur albifrons*)	2	2/0/0	NA	50.0% (1/1/0)	0.0% (0/2/0)	0.0% (0/2/0)	0.0% (0/2/0)	0.0% (0/2/0)	0.0% (0/2/0)	0.0% (0/2/0)	0.0% (0/2/0)	0.0% (0/2/0)	0.0% (0/2/0)	0.0% (0/2/0)	0.0% (0/2/0)	0.0% (0/2/0)	0.0% (0/2/0)	[17]
Proboscidea	Elephantidae	African elephant (*Loxodonta africana*)	20	0/6/14	NA	0.0% (0/20/0)	0.0% (0/20/0)	0.0% (0/20/0)	NA (0/13/7) *	38.5% (5/8/7) *	0.0% (0/20/0)	0.0% (0/20/0)	15.4% (2/11/7) *	7.7% (1/12/7) *	15.4% (2/11/7) *	0.0% (0/20/0)	0.0% (0/20/0)	7.7% (1/12/7) *	15.4% (2/11/7) *	[20,21,25,29,71]
		Asian elephant (*Elephas maximus*)	1	1/0/0	9	100.0% (1/0/0)	0.0% (0/1/0)	0.0% (0/1/0)	0.0% (0/1/0)	0.0% (0/1/0)	0.0% (0/1/0)	0.0% (0/1/0)	0.0% (0/1/0)	100.0% (1/0/0)	0.0% (0/1/0)	100.0% (1/0/0)	0.0% (0/1/0)	0.0% (0/1/0)	0.0% (0/1/0)	[20]
Rodentia	Caviidae	Capybara (*Hydrochoerus hydrochaeris*)	2	1/0/1	NA	0.0% (0/1/1)	0.0% (0/1/1)	0.0% (0/1/1)	0.0% (0/1/1)	0.0% (0/1/1)	0.0% (0/1/1)	0.0% (0/1/1)	0.0% (0/1/1)	0.0% (0/1/1)	0.0% (0/1/1)	0.0% (0/1/1)	0.0% (0/1/1)	0.0% (0/1/1)	0.0% (0/1/1)	[43,69]
	Hystricidae	Crested porcupine (*Hystrix cristata*)	24	0/0/24	NA	0.0% (0/17/7)	0.0% (0/17/7)	0.0% (0/17/7)	0.0% (0/17/7)	0.0% (0/17/7)	0.0% (0/17/7)	0.0% (0/17/7)	0.0% (0/17/7)	0.0% (0/17/7)	0.0% (0/17/7)	0.0% (0/17/7)	0.0% (0/17/7)	0.0% (0/17/7)	0.0% (0/17/7)	[28]
		Total	212	31/24/157		2.0% (4/199/9)	1.5% (3/200/9)	1.0% (2/201/9)	8.7% (17/179/16)	5.6% (11/185/16)	1.0% (2/201/9)	1.0% (2/201/9)	1.9% (3/151/58)	1.5% (3/193/16)	2.0% (4/192/16)	1.5% (3/200/9)	2.0% (4/199/9)	1.5% (3/193/16)	1.0% (2/194/16)	

* Although this pathological finding was reported, the number of affected animals is not available. M = male; F = Female; NA is used to denote the absence of data for a parameter. Cell background color is used to indicate that the postmortem lesion is reported in this species
